# Remote Density Measurements of Molten Salts via Neutron Radiography

**DOI:** 10.3390/jimaging7050088

**Published:** 2021-05-14

**Authors:** Alexander M. Long, S. Scott Parker, D. Travis Carver, J. Matt Jackson, Marisa J. Monreal, Darcy A. Newmark, Sven C. Vogel

**Affiliations:** 1 Materials Science and Technology Division, Los Alamos National Laboratory, Los Alamos, NM 87545, USA; sparker@lanl.gov (S.S.P.); dtcarver@lanl.gov (D.T.C.); jayjacks@lanl.gov (J.M.J.); sven@lanl.gov (S.C.V.); 2 Chemistry Division, Los Alamos National Laboratory, Los Alamos, NM 87545, USA; mmonreal@lanl.gov; 3 Physics Division, Los Alamos National Laboratory, Los Alamos, NM 87545, USA; dnewmark@lanl.gov

**Keywords:** molten salts, thermophysical properties, neutron radiography

## Abstract

With an increased interest in the use of molten salts in both nuclear and non-nuclear systems, measuring important thermophysical properties of specific salt mixtures becomes critical in understanding salt performance and behavior. One of the more basic and significant thermophysical properties of a given salt system is density as a function of temperature. With this in mind, this work aims to present and layout a novel approach to measuring densities of molten salt systems using neutron radiography. This work was performed on Flight Path 5 at the Los Alamos Neutron Science Center at Los Alamos National Laboratory. In order to benchmark this initial work, three salt mixtures were measured, NaCl, LiCl (58.2 mol%) + KCl (41.8 mol%), and MgCl${}_{2}$ (32 mol%) + KCl (68 mol%). Resulting densities as a function of temperature for each sample from this work were then compared to previous works employing traditional techniques. Results from this work match well with previous literature values for all salt mixtures measured, establishing that neutron radiography is a viable technique to measure density as a function of temperature in molten salt systems. Finally, advantages of using neutron radiography over other methods are discussed and future work in improving this technique is covered.

## 1. Introduction

Interest in exploring molten salts has increased within various energy applications due to its observed advantages over other materials, such as high volumetric heat capacity, low vapor pressure, low sensitivity to neutron irradiation, and mutual solubility with actinides [1]. With respect to nuclear applications, the molten salt reactor (MSR) is a heavily pursued advanced reactor concept, with a number of designs and fuel compositions under investigation by developers, both domestically and internationally. In large part, these designs consist of an actinide–halide fuel form dissolved in an alkali/alkaline earth metal halide solvent salt [2,3]. Additionally, a number of designs employ molten halide salts either as a blanket, or strictly as a heat transfer fluid (HTF) [4,5,6]. Beyond MSRs, actinide-bearing chloride salts are used (as either a solvent or electrolyte) in low-enriched uranium recovery for fuel reprocessing of conventional reactor fuel, as well as in plutonium and high-enriched uranium purification processes to produce nuclear material for use in defense programs [7,8,9,10,11,12]. In non-nuclear applications, nitrate molten salts as coolant have been proposed as HTF and in thermal energy storage (TES) systems for concentrated solar power (CSP) systems [13].

With the increasing use of molten salts within these nuclear and non-nuclear systems, knowledge of thermophysical properties becomes ever more relevant in the development of these technologies. As new candidate salts emerge, the ability to measure thermophysical properties, such as density, viscosity, heat capacity, and thermal conductivity, as a function of temperature, pressure, and chemical composition is required to understand and benchmark the material performance within the intended operational conditions. To date, several extensive databases containing thermophysical property values for various molten salts exist with the main goal of providing precise and accurate information on thermophysical properties needed to model the performance of a given salt in both non-nuclear [14,15] and nuclear systems [16]. However, these databases remain incomplete, and additional high quality data are needed.

The density of a given salt as a function of temperature is one of the more significant and fundamental properties needed to better understand the thermophysical behavior, and therefore performance, in a given system. In non-nuclear heat transport systems density is critical in thermal hydrodynamics, and in nuclear systems with liquid salt fuels, density directly impacts the overall reactivity. In the past, various molten salt densities have been measured via traditional techniques, such as the Archimedes’ method, which takes advantage of an object of a known material, volume, and density, that is then suspended in a salt solution. By measuring the apparent mass of the object while fully submerged in the salt, the density of the displaced fluid can be determined. Using a furnace to heat the whole apparatus further allows for densities of the fluid to be determined as a function of temperature. To date, densities of most single-component salt systems have been measured via the Archimedes method (see [14,15,16] and references therein for further review). This method has proven effective, although it requires precise knowledge of the object being suspended in the solution and can be influenced by the presence of voids inside the suspended object or air bubbles on the surface. Additionally, proper material compatibility between the suspended object and the salt solution at elevated temperatures needs to be carefully accounted for as it may complicate the application of this method. This method can be time consuming in terms of measuring the volume of the object, and usually requires several calibration-runs of submerging the object in known solutions before each measurement. More recently, the application of bubbler systems [17,18,19] and liquid surface displacement probes [20] have been developed to measure densities in LiCl + KCl based salt mixtures. Developed for nuclear material accountancy during electrochemical reprocessing, these techniques have proven to be accurate with uncertainties below 1% in terms of density measurements, though there is a need for large amounts of sample materials, on the order of tens to hundreds of grams, which may hinder their application in measuring more exotic salt mixtures where material is sparse.

Given some of the challenges encountered using existing methods, along with large discrepancies often observed in reported results, there is a need to measure and verify densities using a variety of methods. With this in mind we have developed a novel approach to measuring densities of molten salts using neutron radiography. In contrast to the Archimedes’ method, the basic principle behind this method is similar to that of a dilatometer, where the volume is measured of a given liquid or fluid as a function of temperature. This technique has some advantages over other established methods as it is both remote and non-destructive. Additionally, due to the high penetrability of neutrons with more dense materials, imaging of volumes can be performed through complex sample environments, such as bulky furnaces and dense containment vessels. Finally, this method requires relatively small quantities of material (a few grams) compared to other methods, which is useful in the investigation of fissile, actinide-bearing salts, where material maybe limited due to availability or accountability.

## 2. Setup and Methods

Remote density measurements utilizing neutron radiography were performed on Flight Path 5 (FP-5) at the Lujan Neutron Scattering Center, which is one of two spallation neutron sources at the Los Alamos Neutron Science Center (LANSCE, Los Alamos, NM, USA) [21,22]. Located on the lower tier of the Lujan Target-Moderator-Reflector-Shield (TMRS) systems [23], FP-5 observes moderated neutrons from a room temperature high intensity water moderator, with energies ranging from approximately thermal to epithermal (0.25 meV to 10 keV) [24].

### 2.1. Experimental Setup

**Figure 1:**
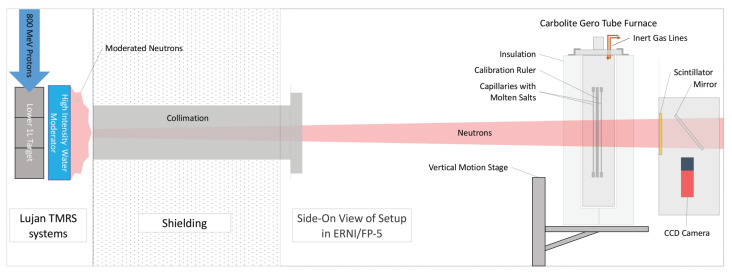
Diagram illustrating a side-on view of the remote density setup on FP-5 at Los Alamos Neutron Science Center (LANSCE) used to measure densities of several molten salts samples at elevated temperatures.

A schematic of the experimental setup for remote density measurements via neutron radiography is illustrated in Figure 1. Here, moderated neutrons coming from one of the high intensity water moderators within the TMRS system travel down the flight path where they are collimated through an alternating borated polyethylene and steel collimator. The collimation starts at roughly 2 cm in diameter and gradually opens to 4 cm over a length of 1.2 m. A resistive heating tube furnace from Carbolite Gero (Sheffield, UK) was placed 3.71 m downstream from the end of the final collimator. Placed within the furnace was a ISO 63 containment vessel housing two salt samples sealed in 0.25” stainless steel tubes with Swagelok caps on either side along with a sample holder that also acts as calibration ruler. All components were made out of either 304 or 316 stainless steels which are important to note for including coefficients of thermal expansion when calculating sample volumes at elevated temperatures. A continuous slow flow of argon was used as an inert cover gas to prevent oxidation at higher temperatures. Within the furnace, temperatures were continuously monitored inside the containment vessel using a K type thermocouple attached to a 332 Lakeshore temperature readout. Additionally, temperature uniformity within the containment vessel was measured prior to density measurements using five thermalcouples placed at various heights inside the vessel. These initial tests found all five thermocouple readings to be within a 5 ${}^{\circ }$C range across all temperatures tested. Finally, the whole furnace setup was placed on a motion stage to allow for vertical scanning of the samples.

Located roughly 50 cm downstream of the samples and furnace was the neutron imaging camera, which consisted of a 20 μm thick Gadolinium oxysulfide (Gd${}_{2}$O${}_{2}$S) scintillator screen with a 10 cm × 10 cm field-of-view, a thin walled mirror, a NIKOR 200 mm lens (Tochigi, Japan), and an ATIK 490 ex CCD camera (Norwich, UK), all of which was enclosed in a light-tight aluminum box. The resolution of the entire neutron imaging setup, as observed at the position of the Gd${}_{2}$O${}_{2}$S scintillator screen, was measured using a United States Air Force (USAF) 1951 test target and was determined to be approximately 25 microns/pixel.

### 2.2. Sample Selection and Preparation

In total, three molten salt samples were selected to measure: single-component NaCl, along with the eutectic mixtures of both LiCl-KCl and MgCl-KCl. Sample mixtures were chosen for these initial measurements because the density as a function of temperature for each sample has been well characterized and reported in previous works. All samples measured in this work are listed in Table 1.

For these measurements samples were prepared off site from LANSCE within an inert atmosphere glove box, where moisture and oxygen were controlled. High purity (>99.99%) chloride salts were purchased from Sigma-Aldrich and were subsequently dried by an established vacuum furnace method. All samples were characterized by X-ray powder diffraction to confirm purity.

Once prepared, dried salt samples were placed and sealed inside a 0.25” thick, ∼9” tall stainless steel (316 SS) tube with Swagelok caps on either side, all within an inert atmosphere. Masses of each salt sample were obtained by measuring the mass of the 0.25” thick Swagelok tubing with and without the salt samples on an analytical balance multiple times. Once sealed, samples were then attached on either side of a fabricated stainless steel (304 SS) holder that also acted as a ruler with notches of known lengths along the spine of the holder. Two samples at a time, along with the holder, were then placed inside the containment vessel to be heated and radiographed.

### 2.3. Imaging

With a field of view of ∼9 × 9 cm${}^{2}$, multiple exposures while adjusting the vertical motion stage were needed to cover the complete height of the samples. In order to cover a height of roughly 9 inches, seven overlapping images were taken for each sample pair. Each image was averaged over 4–6 five-minute exposures. At the beginning of each measurement, samples were heated within the furnace to the maximum test temperature (usually around 1020 ${}^{\circ }$C) in order to release any potential bubbles produced in the melting process. While holding the samples at the maximum temperature, a full scan was performed from top to bottom in order to locate the menisci in both samples and to identify any potential bubbles or abnormalities within the fluids. Once located, the field of view is centered around the menisci in each sample. Images were then taken of the meniscus at specific temperatures as the sample is cooled. The full scan of the samples at maximum temperatures, plus images of the meniscus as the sample cools allows for the total volume of the fluids to be calculated at each temperature measured.

### 2.4. Image Analysis and Volume Calculations

**Figure 2:**
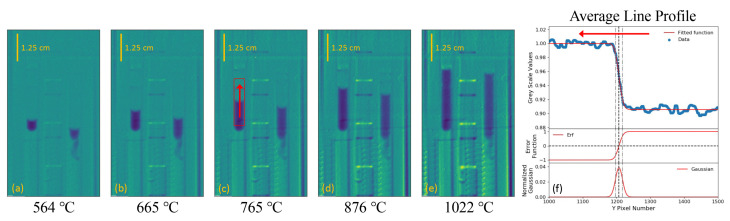
(**a**–**e**) Images of LiCl + KCl eutectic samples at varying temperatures normalized to the 463 ${}^{\circ }$C image with highly adjusted contrast. (**f**) A plot of the pixel grey scale value as a function of the y-pixel height, averaged over the region highlighted in red shown in (**e**). These grey scale values are fitted with a normal distribution error function (Equation (1)) to extract the centroid and standard deviation for the location of the meniscus in the given image. It should be noted here that a slight tail structure can be observed in the right-hand sample. This structure, while not directly observed in the image taken at 463 ${}^{\circ }$C, is highlighted with this ratio method.

In order to determine the total volume of the fluid as a function of temperature, the location of the meniscus for each sample needs to be accurately measured. This would traditionally be done using transmission images by taking the ratio of an image with the samples in vs the image with the samples out, where contrast due to the sample can be isolated from the surrounding background and the location on the meniscus can easily be measured. Due to the weight of the containment vessel and the slack in the furnace stand, proper transmission images could not be obtained with this setup as even the slightest movement of the furnace would create large background artifacts. Instead, meniscus levels were determined by taking the ratio of an image at one temperature to an image at another, usually the lowest temperature recorded. This method allows for a more precise extractions of meniscus locations for the two samples at both of the relative temperatures by normalizing out much of the observed background structures within the furnace. This technique, shown in Figure 2, proved to be useful in determining meniscus heights in all samples even ones with lower contrast.

The exact position of the meniscus in a given sample image was determined by fitting an averaged pixel grey value line profile over the width of the sample with the following error function of a normal distribution,

(1) $$G\left(y\right)=A\mathrm{erf}\;\left(\frac{y-y_{0}}{\sqrt{2}\sigma }\right)$$

Here, *G(y)* is the pixel grey value profile as a function of pixel height, *A* is the absolute magnitude of the error function, *y*${}_{0}$ is the centroid of the normal distribution, and σ is the standard deviation. An example of taking the ratio of images to isolate the change in the meniscus heights with LiCl + KCl eutectic samples, along with fitting line profiles with the error function of a normal distribution, is shown in Figure 2. Here, averaged grey scale values were taken over the width of the sample, illustrated as a red box in Figure 2, in order to create smooth line profiles for fitting. Once fitted, the centroid, *y*${}_{0}$, is taken at the height of the meniscus, and the standard deviation, σ, is used as the uncertainty in the meniscus height.

**Figure 3:**
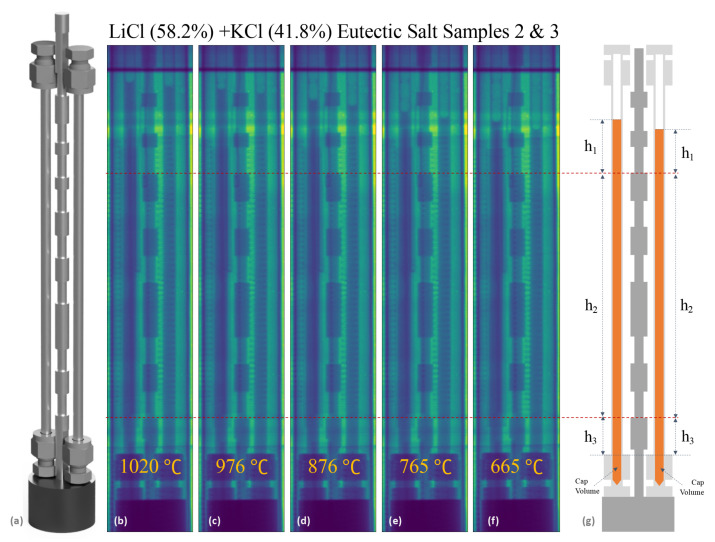
(**a**) A CAD rendering of 0.25” inch OD Swagelok tube containers along with the sample holder that acts as a calibration ruler. (**b**–**f**) Composite radiographs of two LiCl + KCl eutectic samples (2 and 3) taken at various temperatures 1020–665 ${}^{\circ }$C). (**g**) Diagram illustrating how heights of menisci are determined relative to the sample holder.

In order to determine the total volume of the fluid as a function of temperature, the location of the meniscus in each sample is measured relative to the nearest notch on the sample holder, thus allowing for the overall heights in each fluid to be determined. To illustrate this, a computer-aided design (CAD) rendering of the salt containing sample tubes, plus the sample holder is shown in Figure 3a, along with stitched composite radiographs of two LiCl + KCl eutectic samples taken at various temperatures (Figure 3b–f). Due to the possibility of sample tubes not being in complete contact with the base of the reference, heights of the fluids were broken down into three measurements: the height of the meniscus to the nearest notch (h${}_{1}$), the height of the sample holder from the notch nearest the meniscus to the bottom most notch (h${}_{2}$), and the bottom most notch to the top of the Swagelok cap (h${}_{3}$). Finally, with the geometry of the fluid within the bottom most Swagelok cap taken from manufacturing specifications, a total height can be determined for each sample (illustrated in Figure 3g).

With the total height determined, manufacture specifications of the inner diameter of the Swagelok tubing (i.d. = 4.572 ± 0.1 mm) along with the assumption of axial symmetry were used to calculate fluid volumes. Additionally, given that fluid height measurements were performed at high temperatures (up to 1023 ${}^{\circ }$C), volume changes due to thermal expansion within the 316 SS tubing and the 304 SS sample holder need to be accounted for. Therefore, the coefficients of thermal expansion (CTE) for these two steels were measured independently by a force-controlled push-rod dilatometer. The CTE was determined by the ratio method described in ASTM standard E473011a, with reference to the known expansion of Al${}_{2}$O${}_{3}$. All CTE measurement were conducted under flowing argon environment in order to limit the apparent oxidation of the steel samples. Measurements from the 304 SS samples resulted with a CTE of 20 × (T/10${}^{6}$) − 0.007, and measurements of the 316 SS samples gave a CTE of 19.8 × (T/10${}^{6}$) − 0.007, where T is in units of K. Once measured, CTEs were applied to the respective materials (sample holder, Swagelok tubing and cap) to get an accurate geometries and therefore volume measurements of samples at elevated temperatures. Finally, with the total mass of each sample determined prior to being placed inside the furnace and the volumetric measurements obtained with neutron radiography, densities for all samples were obtained as a function of temperature.

## 3. Results and Discussion

**Figure 4:**
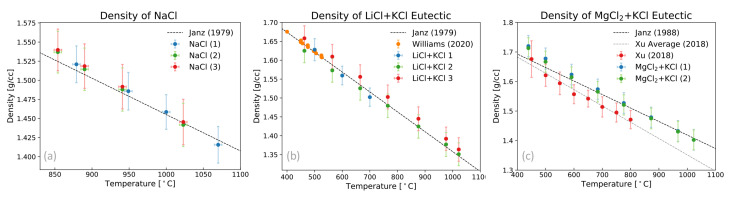
Density measurements via neutron radiography from this work as a function of temperature, along with selected previous works, for (**a**) NaCl, (**b**) LiCl + KCl eutectic, and (**c**) MgCl${}_{2}$ + KCl eutectic samples.

Utilizing neutron radiography techniques with the setup detailed above, menisci heights were measured as a function of temperature for three salt mixtures, NaCl, LiCl + KCl (eutectic), and MgCl${}_{2}$ + KCl (eutectic). These height measurements, along with relatively simple assumptions on the geometry of the sample tubes and mass measurements, were then used to determine sample densities as a function of temperature. The final density results from this neutron radiography measurement for each of the chosen samples are shown in Figure 4. Starting with results for NaCl, shown in Figure 4a, density measurements from this work were compared to previous literature values [14,25,26]. In this work, densities of three NaCl salt samples were measured and show good agreement with previous works. Similarly, density measurements of three LiCl + KCl eutectic samples were measured and compared to evaluated values from Janz et al. [14,15,27], along with a more recent measurement of the salt system using a triple bubbler setup [17]. All three LiCl + KCl density measurements from this work agree within their uncertainties with the previous measurements and evaluations. The final salt mixture measured in this work was the MgCl${}_{2}$ + KCl eutectic system. This system was chosen for this work as it was recently measured by Xu et al. [28] using the Archimedes method, and showed slight deviations from evaluated literature [14]. In this work, density measurements were performed on two different samples and show agreement within error of the quote values of Xu et al. and evaluated Janz et al. over the temperature range measured, though there is some amount of discrepancy between the average values quoted by Xu et al. and the measurements from this work.

In most of the measurements, the final uncertainty in density is on the order of 1–3%. The main contribution to this final uncertainty is the inner diameter of the Swagelok tubing, as small variations in the wall thickness can be compounded along the length of the sample. Given the potential for variations in the wall thickness from one tube to the next (and within each tube), uncertainties can be reduced either by calibrating the inner diameter of specific individual tubes using a liquid with a known density, such as high purity water, to get an effective inner radius, or by rotating the sample during neutron radiography measurements and using computed tomography (CT) reconstruction techniques to obtain a comprehensive measurements of the total inner volume. With the ability to reduce the uncertainty in volume associated with radius, this method has the potential for direct measurements of density with 0.6–0.8% uncertainty. Additionally, there is some amount of uncertainty in the line profile fitting of the meniscus, where the assumption of a flat surface is made. Some samples actually show a significant curvature in the meniscus shape or even small artifacts, as seen for the LiCl + KCl eutectic measurements in Figure 3 and in Figure 2, which can result in larger uncertainties for those density measurements. Taking into account potential changes in the curvature in the meniscus as a function of temperature, the later suggestion of performing CT would greatly improve the final uncertainty in these types of density measurements as one can integrate over all voxels containing the sample material to obtain a very precise volume as a function of temperature. Furthermore, these final uncertainties would only depend on the mass measurement, pixel pitch, and number of rotations in the CT scan. With this in mind, future work is planned on FP5 to enable CT-reconstruction of sample volumes by designing samples that can rotate within the furnace.

It should be noted that density measurements of molten salts utilizing neutron radiography is a novel application that is still under development. Currently other techniques, such as the Archimedes method or triple bubbler system, result in final density measurements with lower uncertainties, though there is great potential for this neutron radiography method to improve upon these initial measurements with slight modifications. From this work, it is clear that there are certain advantages to using neutron radiography methods over other traditional methods. The first is the ability to measure samples remotely with the high penetrability of neutrons, which ultimately allows potential volume characterizing in more exotic sample environments, such as large furnaces capable of reaching extreme temperatures, or materials that require multiple levels of containment such as Pu-based salts or irradiated salts. Additionally, the remote and non-destructive nature of neutron radiography allows for further thermophysical measurements of the exact sample afterwards, thus increasing the efficiency of information extracted from any given sample. Another advantage of neutron radiography is the unique potential to measure densities on salts that are limited by the total amount of material available. This type of capability directly impacts measurements of salt mixtures such as those containing plutonium where only a few grams are currently available. Here, great care can be taken when designing a container to house the salts and enhance any volumetric changes as a function of temperature. Additionally, this technique allows for direct observation of the entire sample throughout the measurement. This information is helpful in identifying defects within the sample which many alter resultant measurements, such as bubbles or partially melted solutions, and has the potential to identify density variations due to any temperature differences across the sample, thus increasing the confidence levels in the final density results. Furthermore, this technique can measure multiple samples simultaneously, and the maximum number of samples only depends on the size of the neutron beam, the size and effectiveness of the furnace, and the field of view of the neutron imaging detector. Finally, this technique has been shown in this work to cover larger temperature ranges when compared to previous measurements with other techniques.

In addition to the samples and results presented in this work, this neutron radiography technique has been applied to measuring densities in a comprehensive set of NaCl + X mol% uranium trichloride (UCl${}_{3}$) samples and will be reported elsewhere. Ultimately, density measurements via neutron radiography show great potential as an additional and alternative method for molten salt systems, along with other systems where a volumetric expansion is significant enough to spatially measure via imaging. Additionally, further work is underway on FP5 at LANSCE to explore and improve this type of neutron radiography technique such that this method can be more widely adopted as a potential tool.

## Figures and Tables

**Table 1:** List of samples used for density measurements with neutron radiography on FP5 at LANSCE, along with the mass of each sample, the mole fraction, melting point, and temperature range over which the density measurements were made.

Material	Mass	Mole	Melting	Temperature
(Sample ID)	[g]	Fractions	Point [${}^{\circ }$C]	Range [${}^{\circ }$C]
NaCl (1)	3.8951(5)	NaCl: 100%	801	879–1070
NaCl (2)	4.7984(5)	NaCl: 100%	801	854–1023
NaCl (3)	4.7906(5)	NaCl: 100%	801	854–1023
LiCl + KCl (1)	4.0493(5)	LiCl: 58.2%, KCl: 41.8%	355	500–700
LiCl + KCl (2)	4.4957(5)	LiCl: 58.2%, KCl: 41.8%	355	463–1025
LiCl + KCl (3)	4.5003(5)	LiCl: 58.2%, KCl: 41.8%	355	463–1025
MgCl${}_{2}$ + KCl (1)	4.2896(5)	MgCl${}_{2}$: 32%, KCl: 68%	430	439–1023
MgCl${}_{2}$ + KCl (2)	4.1571(5)	MgCl${}_{2}$: 32%, KCl: 68%	430	439–1023

## Data Availability

Not applicable.
